# PKA regulates autophagy through lipolysis during fasting

**DOI:** 10.1016/j.mocell.2024.100149

**Published:** 2024-11-13

**Authors:** Yul Ji, Yong Geun Jeon, Won Taek Lee, Ji Seul Han, Kyung Cheul Shin, Jin Young Huh, Jae Bum Kim

**Affiliations:** 1 Center for Adipocyte Structure and Function, Institute of Molecular Biology and Genetics, School of Biological Sciences, Seoul National University, Seoul, South Korea; 2 Department of Life Science, Sogang University, Seoul, South Korea

**Keywords:** AMP-activated protein kinase, Autophagy, *Caenorhabditis elegans*, Free fatty acid, Lipolysis, Protein kinase A

## Abstract

Autophagy is a crucial intracellular degradation process that provides energy and supports nutrient deprivation adaptation. However, the mechanisms by which these cells detect lipid scarcity and regulate autophagy are poorly understood. In this study, we demonstrate that protein kinase A (PKA)-dependent lipolysis delays autophagy initiation during short-term nutrient deprivation by inhibiting AMP-activated protein kinase (AMPK). Using coherent anti-Stokes Raman spectroscopy, we visualized free fatty acids (FFAs) in vivo and observed that lipolysis-derived FFAs were used before the onset of autophagy. Our data suggest that autophagy is triggered when the supply of FFAs is insufficient to meet energy demands. Furthermore, PKA activation promotes lipolysis and suppresses AMPK-driven autophagy during early fasting. Disruption of this regulatory axis impairs motility and reduces the lifespan of *Caenorhabditis elegans* during fasting. These findings establish PKA as a critical regulator of catabolic pathways, prioritizing lipolysis over autophagy by modulating AMPK activity to prevent premature autophagic degradation during transient nutrient deprivation.

## Graphical abstract

**Figure:**
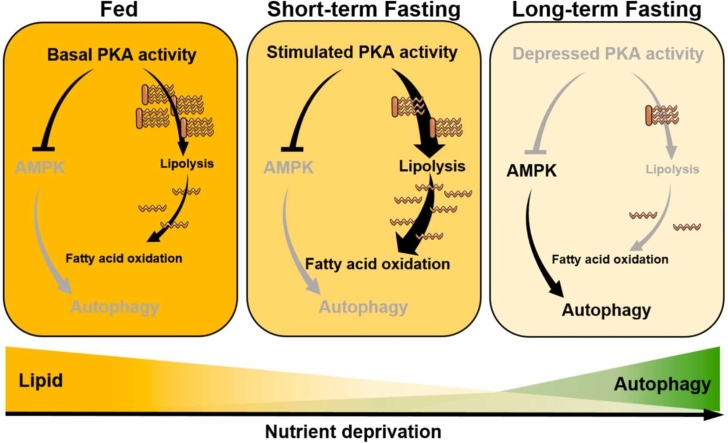

## INTRODUCTION

The most common challenge for all living organisms is sensing and responding to nutritional deprivation to survive. Under severe nutrient deprivation, eukaryotic cells evolve to initiate macroautophagy (hereafter referred to as autophagy), during which cellular components are degraded to obtain energy sources for survival (Palm and Thompson, 2017). Although many studies have shown that nutrient deprivation (i.e., depletion of glucose or amino acids) induces autophagy (Russell et al., 2014), it remains unclear when and how autophagy is initiated by sensing insufficient intracellular energy sources. In addition, autophagy does not appear to occur immediately after nutrient deprivation because most animals reserve extra energy sources, such as lipids, for short-term starvation to prevent nonselective and self-destructive autophagy for survival.

During nutrient deprivation, autophagy initiation is tightly regulated by several kinases that recognize the intracellular energy state. For example, AMP-activated protein kinase (AMPK) promotes autophagy under low-energy conditions (Egan et al., 2011, Hardie, 2007), whereas the mammalian target of rapamycin complex 1 inhibits autophagy under high-energy conditions (Gleason et al., 2007, Kim et al., 2011). ULK1, VPS34, and other autophagy-related genes (ATGs), which are downstream targets of AMPK and mammalian target of rapamycin complex 1 (mTORC1), guide stepwise autophagy progression from phagophore formation to lysosomal fusion (Zachari and Ganley, 2017). Upon activation of ULK1, the VPS34 complex containing Beclin-1 and ATG14 is recruited to the phagophore, followed by sequential autophagy processes such as sequestration, transport to lysosomes, and degradation (Russell et al., 2014, Vainshtein and Hood, 2016). Although enzymatic activities in each autophagic process have been investigated under amino acid or glucose deprivation conditions (Kishi-Itakura and Buss, 2018, Sengupta et al., 2010, Song et al., 2023), the physiological circumstances that accompany the reduction of major energy storage products such as lipids have not been thoroughly examined. Considering that the extent of nutrient reduction for autophagy initiation is predicted to vary across cell or tissue types (Pfeifer and Strauss, 1981, Mortimore and Poso, 1987, Mizushima et al., 2004), the degree and type(s) of nutrient deprivation, particularly lipids that initiate autophagy for survival remain unclear.

Although autophagy mediates beneficial roles in survival under cellular stress conditions (Murrow and Debnath, 2013), unnecessary initiation of autophagy can have deleterious effects (Herath et al., 2024). For example, excessive or sustained autophagic degradation of cellular components results in muscular dystrophy (Bonaldo and Sandri, 2013, Malicdan et al., 2008), reduced insulin storage (Yamamoto et al., 2018), and even cell death (Liu and Levine, 2015), indicating that tight temporal regulation of autophagy initiation is critical for physiological homeostasis. Furthermore, autophagy is considered the cell's last defense process to meet energy demands because of its self-degrading nature (Gladkova et al., 2018). Autophagy initiation likely only occurs when proper energy sources are unavailable. Thus, it is important to understand the regulatory mechanisms(s) that switch autophagy “on/off” with respect to intracellular nutrient availability under dynamic nutrient deprivation conditions.

## MATERIALS AND METHODS

### *Caenorhabditis elegans* Culture and RNA Interference

All worm strains were grown at 20°C on nematode growth medium plates seeded with *Escherichia coli* (OP50) as a food source unless otherwise mentioned. The wild-type strain used was Bristol strain N2. The *Caenorhabditis* Genetics Center provided hjIs67[atgl-1p::atgl-1::gfp] and adIs2122[lgg-1p::gfp::lgg-1] worms. When synchronization of developmental stages was necessary, the animals were lyzed to prepare the eggs according to the standard protocol. After overnight incubation, synchronized L1 stage worms were plated on food; worms in the young adult stage were collected after 40 ± 2 hours for either molecular or behavioral analyses. For the feeding RNA interference (RNAi) experiments, *kin-1*, *kin-2*, *aak-2*, *agtl-1*, *cpt-2*, *cpt-3*, and *acs-2* RNAi clones were obtained from the Ahringer and Vidal RNAi libraries. Synchronized L1 stage worms were cultured on RNAi plates until they reached the young adult stage. The RNAi efficiency was confirmed by reverse transcription PCR using appropriate primers.

### *Caenorhabditis elegans* Assays

For lifespan assays, synchronized worms were grown and maintained on nematode growth medium plates. The numbers of live and dead worms were counted daily until no live worms remained. To measure bending rates, the worms were suspended in M9 buffer, and their movements were analyzed. The pumping rates were manually counted by measuring the number of contraction and relaxation cycles of the pharyngeal muscles using bright-field imaging with a confocal microscope.

### *Caenorhabditis elegans* Neutral Lipids Staining

A BODIPY 493/503 (D2933, Thermo Fisher Scientific) stock solution (1 mg/ml) and diluted (1:200) LipidTOX (H34477, Invitrogen) were prepared in dimethyl sulfoxide. For fixative staining, the worms were incubated in 4% paraformaldehyde solution for 15 minutes, followed by 3 freeze/thaw cycles in liquid nitrogen. After removing the paraformaldehyde solution by washing with M9, the worms were incubated in 500 µl of 1 µg/ml BODIPY 493/503 or diluted LipidTOX in M9 for 1 hour at room temperature. The worms were washed with M9 buffer and used for microscopic imaging.

### *Caenorhabditis elegans* Lysotracker Staining

The worms were transferred to solid media and spread with 1:1,000 diluted LysoTracker (L7526 or L12492, Thermo Fisher Scientific). After 1 hour in the dark, the worms were washed extensively with phosphate-buffered saline to remove the dye and used for microscopic imaging.

### Cell Line Experiments

3T3-L1 preadipocytes were grown in Dulbecco’s modified Eagle’s medium (DMEM) supplemented with 10% bovine calf serum. Two days after reaching confluence, the cells were induced to differentiate into adipocytes in DMEM containing 10% fetal bovine serum (FBS), 3-isobutyl-1-methylxanthine (520 μM), dexamethasone (1 μM), and insulin (167 nM) for 48 hours. The differentiation induction medium was replaced with DMEM containing 10% FBS and insulin (167 nM). After 2 days, the medium was replaced with DMEM containing 10% FBS every 2 days for 5 to 7 days. To visualize neutral lipids in 3T3-L1 adipocytes, BODIPY 493/503 stock solution was diluted 1:1,000 in DMEM containing 10% FBS and incubated with the cells for 30 minutes.

### Coherent Anti-Stokes Raman Spectroscopy Imaging for Lipids

Using a Leica TCS SP8 multiphoton microscope, the output of an optical parametric amplifier (APE) was used as the excitation source for the pump and Stokes beams. Images were recorded at a laser frequency of 2,854 cm^−1^, corresponding to the aliphatic CH_2_ vibrational resonance. The pump source of the optical parametric oscillator was tuned to 670 nm to achieve resonance. The beams were focused onto the object plane using a 40×/NA water-immersion objective, which was used for the coherent anti-Stokes Raman spectroscopy (CARS) and fluorescence measurements. The powers of both the Stokes pulse and pump were approximately 100 mW.

### Quantification of CARS-Only Signals

The intensities of total CARS and neutral lipid signals were quantified using LAS X (Leica software). ImageJ software (NIH) was used for CARS-only signal measurements. Pseudo-colored CARS total signals (blue) and neutral lipid signals (red) were split into 3 channels (red, blue, and green), followed by subtraction of the red channel of the neutral lipid signal image from the blue channel of the CARS total signal image.

### Western Blotting

Cells were lyzed on ice using a modified RIPA buffer containing 50 mM Tris-HCl (pH 7.5), 150 mM NaCl, 2 mM EDTA, 1% (v/v) Triton X-100, 0.5% (w/v) sodium deoxycholate, 0.1% (w/v) sodium dodecyl sulfate, 5 mM NaF, 1 mM Na_3_VO_4_, and a protease inhibitor cocktail (P3100, GeneDEPOT, Altair). Antibodies against the pPKA substrate (9624S, Cell Signaling Technology; 1:1,000), GAPDH (LF-PA0018, LabFrontier; 1:1,000), pAMPK (2531, Cell Signaling Technology; 1:1,000), AMPK (2532, Cell Signaling Technology; 1,000), LC3B (NB100-2220, Novus Biologicals; 1:1,000), P62 (H00008878-M01, Novus Biologicals; 1:1,000), GFP (sc-9996, Santa Cruz Biotechnology; 1:1,000), actin (ab14128, Abcam; 1:2,000), and β-actin (A5316, Sigma-Aldrich; 1:2,000) were used for western blotting analysis. The bands were visualized using horseradish peroxidase–conjugated secondary anti-rabbit or anti-mouse IgG antibodies (A0545 and A9044, respectively; Sigma-Aldrich).

### Quantitative Reverse Transcription-Quantitative Polymerase Chain Reaction

Total RNA was isolated using the TRIzol reagent (Invitrogen) according to the manufacturer’s instructions. cDNA was synthesized using a reverse transcriptase kit (Thermo Fisher Scientific) according to the manufacturer’s instructions. Primers used for reverse transcription-quantitative polymerase chain reaction were obtained from Bioneer.

### Metabolome Profiles of *C. elegans* Using Capillary Electrophoresis and Liquid Chromatography Time-of-Flight Mass Spectrometry Analysis

The worm pellets from the experimental groups were freeze-dried overnight and stored at room temperature until further extraction. Samples were analyzed using capillary electrophoresis time-of-flight mass spectrometry (CE-TOF-MS) and liquid chromatography time-of-flight mass spectrometry (LC-TOF-MS) (Human Metabolome Technologies, Inc.). Metabolomic profiling of 9 *C. elegans* samples revealed 264 metabolites using CE-TOF-MS (160 in cationic mode and 104 in anionic mode) and 88 metabolites using LC-TOF-MS (44 in positive mode and 44 in negative mode). For CE-TOF-MS analysis, the samples were mixed with 50% acetonitrile in water (v/v) containing internal standards, homogenized, and filtered through a 5 kDa cutoff filter to remove macromolecules. The filtrate was concentrated and resuspended in ultrapure water before measurements. For LC-TOF-MS analysis, the samples were mixed with 1% formic acid in acetonitrile (v/v) containing internal standards, homogenized, centrifuged, and filtered twice to remove proteins and phospholipids. The final filtrate was then desiccated and resuspended in 50% isopropanol. The metabolites were measured in cationic and anionic modes (CE-TOF-MS) and positive and negative modes (LC-TOF-MS). Peaks were extracted using MasterHands software to obtain the *m/z*, migration time (MT), retention time (RT), and peak area. Peak areas were normalized using internal standards, with the detection limit set at a signal-to-noise ratio of 3. Putative metabolites were assigned based on *m/z*, MT, and RT values, with tolerance ranges of ±0.5 minutes for MT, ±0.3 minutes for RT, ±10 ppm for CE-TOF-MS, and ±25 ppm for LC-TOF-MS. Absolute quantification of the target metabolites was performed using standard curves and internal standard normalization. Additional procedures were described previously (Soga et al., 2002, Soga et al., 2003).

### Statistical Analysis

Data are expressed as the mean ± SD. The means of the 2 groups were compared using a 2-tailed Student’s *t*-test. The means of multiple groups were compared using a 1-way analysis of variance followed by Tukey’s post hoc test. Two independent variables were compared using a 2-way analysis of variance, followed by Sidak’s multiple comparison test. Statistical analyses were performed using GraphPad Prism software (GraphPad Software).

## RESULTS

### Autophagy Is Initiated by Long-Term Nutritional Deprivation

**Fig. 1:**
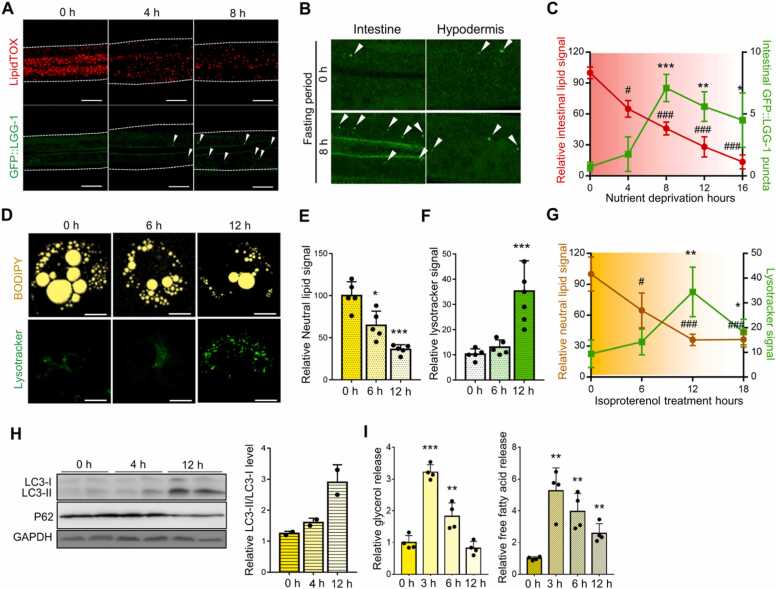
Autophagy is initiated by long-term nutritional deprivation. (A) In worms, lipids and autophagy initiation were visualized over time during fasting. Representative images of lipids and GFP::LGG-1 puncta (white arrowhead). (B) Increased GFP::LGG-1 puncta (white arrowhead) during fasting in the intestine. (C) Time course measurement to quantify lipid levels based on LipidTOX signal and intestinal GFP::LGG-puncta during fasting (n = 7). (D-F) 3T3-L1 adipocytes were treated with isoproterenol (1 μM). BODIPY signal was used for neutral lipid measurement (E), and lysosomal activity was observed by lysotracker staining (F). (G) Measurement of neutral lipids and lysotracker signal over time during fasting (n = 5). (H) Levels of LC3 and P62 protein were detected by western blotting in 3T3-L1 adipocytes treated with isoproterenol (1 μM). (I) Relative levels of glycerol and free fatty acids in 3T3-L1 adipocytes upon isoproterenol (1 μM) treatment. Data represent the mean ± SD; *P < .05, **P < .01, ***P < .001 vs 0-hour fasted group (or isoproterenol-treated group), ^#^*P* < .05, ^###^*P* < .001 vs 0-hour fasted group (or isoproterenol-treated group).

*Caenorhabditis elegans* is an excellent model organism for studying energy metabolism because it enables the imaging of metabolites such as lipids (Ashrafi et al., 2003, Shi et al., 2018). Consistent with previous reports (Lee et al., 2014, McKay et al., 2003), anterior intestinal lipid levels decreased after 4-hour fasting (Fig. 1A). Furthermore, GFP-tagged LGG-1 (nematode ortholog of Atg8/LC3) puncta, a marker of autophagy initiation, was primarily detected in the intestine after 8-hour fasting (Fig. 1A and B), indicating that autophagy was not initiated during short-term fasting (STF) and was accompanied by a decrease in lipid levels during long-term fasting (LTF) (Fig. 1C). Additionally, mammalian adipocytes stimulated with the beta-adrenergic receptor agonist isoproterenol (Zhou et al., 2018) showed increased autophagic activity signals after reductions in lipid droplets sizes (Fig. 1D-G) and lipolytic activity (Fig. 1I). The levels of LC3-II were elevated according to western blot analyses, consistent with the significant reduction in neutral lipid signals (Fig. 1H). Thus, autophagy initiation may be inversely correlated with the stored lipid content.

### Autophagy Is Initiated When Stored Lipid Levels are Decreased During Fasting

**Fig. 2:**
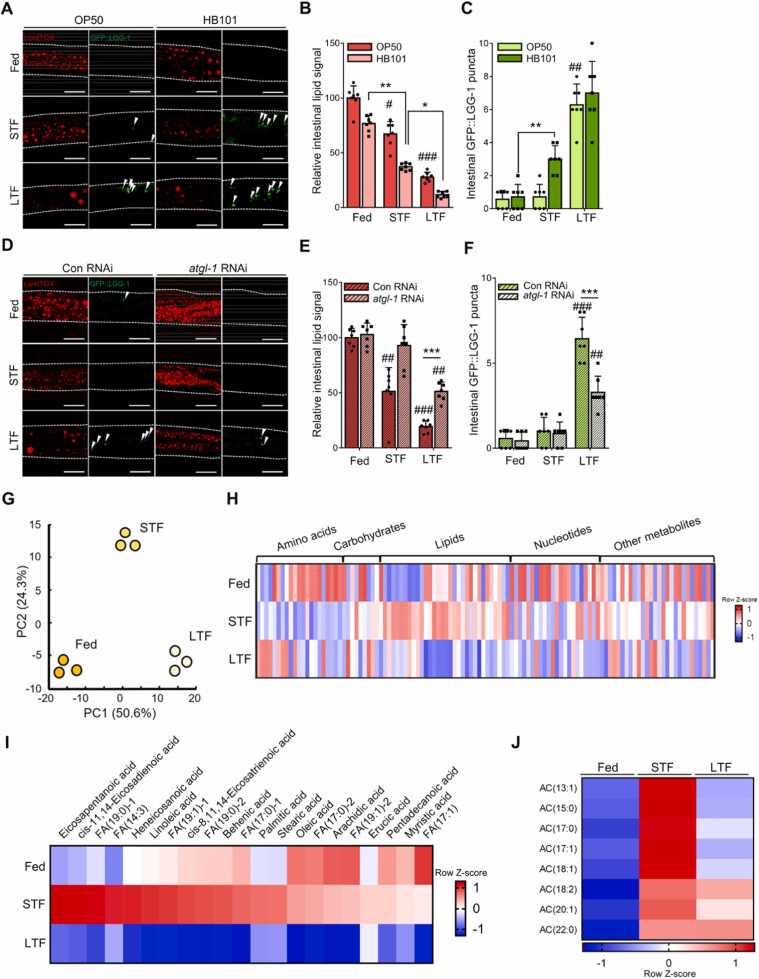
Autophagy is initiated when stored lipids are decreased during fasting. (A-C) Worms were fed with different sources of bacteria, followed by fasting. Representative images of lipids and GFP::LGG-1 puncta (white arrowhead) (A), quantification data of intestinal lipids (B), and GFP::LGG-1 puncta (C) during short-term fasting (STF, 4 hours) and long-term fasting (LTF, 8 hours; *n* = 7). (D–F) *atgl-1* was suppressed via feeding RNA interference (RNAi), resulting in attenuated lipids breakdown throughout fasting (STF [4 hours], LTF [8 hours]). Fewer decreased lipids during LTF (E) with attenuated increased GFP::LGG-1 puncta (F) (*n* = 7). (G) Principal component (PC) analysis plot of the first 2 principal components (PC1 and PC2) generated from the metabolome profiles of worm extracts during fasting (STF [4 hours], LTF [8 hours]). (H) Heat map of metabolites in metabolomic analysis (STF [4 hours], LTF [8 hours]). (I and J) Heat map of fatty acids (I) and acylcarnitines (J) in metabolomic analysis (STF [4 hours]), LTF [8 hours]). All scale bars are 10 µm. Data represent the mean ± SD; *P < .05, **P < .01, ***P < .001, and ^#^*P* < .05, ^##^*P* < .01, ^###^*P* < .001 vs fed.

To investigate whether autophagy and a decrease in stored lipid levels occur independently during fasting or whether the amount of stored lipids affects the time of autophagy initiation, we designed several experimental groups with different amounts of stored lipids during fasting. We fed *C. elegans* different bacterial diets, OP50 and HB101. HB101-fed worms exhibited relatively lower levels of stored lipids than did OP50-fed worms (Fig. 2A), which is consistent with a previous report (Brooks et al., 2009). As shown in Figure 2B, HB101-fed worms exhibited lower levels of stored lipid signals than did OP50-fed worms throughout the fasting period. In HB101-fed worms, GFP:LGG-1 puncta signals were greatly elevated during STF (Fig. 2C), suggesting that low levels of stored lipids are closely associated with autophagy initiation. To confirm this result, *atgl-1*, a major lipase produced during fasting (Zhou et al., 2018) in *C. elegans* (Lee et al., 2014), was suppressed by RNAi to decelerate the breakdown of stored lipids. During fasting, *atgl-1*-suppressed worms (*atgl-1* RNAi) showed a less pronounced reduction in lipid signals than did control worms (Fig. 2D and E). Furthermore, the increase in GFP::LGG-1 puncta signals in control worms, which is typically observed during LTF, was significantly suppressed in *atgl-1*-knockdown worms (Fig. 2F). Metabolomic analysis (Table S1) further revealed that worms at different nutrient deprivation periods had distinct metabolite profiles (Fig. 2G), with decreased lipid levels in the LTF group (Fig. 2H). Although carbohydrate and amino acid levels appeared to vary during different fasting periods (Fig. S1A), the increased free fatty acid (FFA) and acylcarnitine levels in the STF group tended to decrease in the LTF group (Fig. 2I and J). Considering the increased expression of fatty acid oxidation-related genes during STF (Fig. S1B), FFAs generated during the early phase of fasting may be consumed before LTF. These data suggest that autophagy initiation occurs at a relatively later phase of nutritional deprivation than when the stored lipids are acutely hydrolyzed.

### Autophagy Is Associated With Decreased FFA Levels During Fasting

**Fig. 3:**
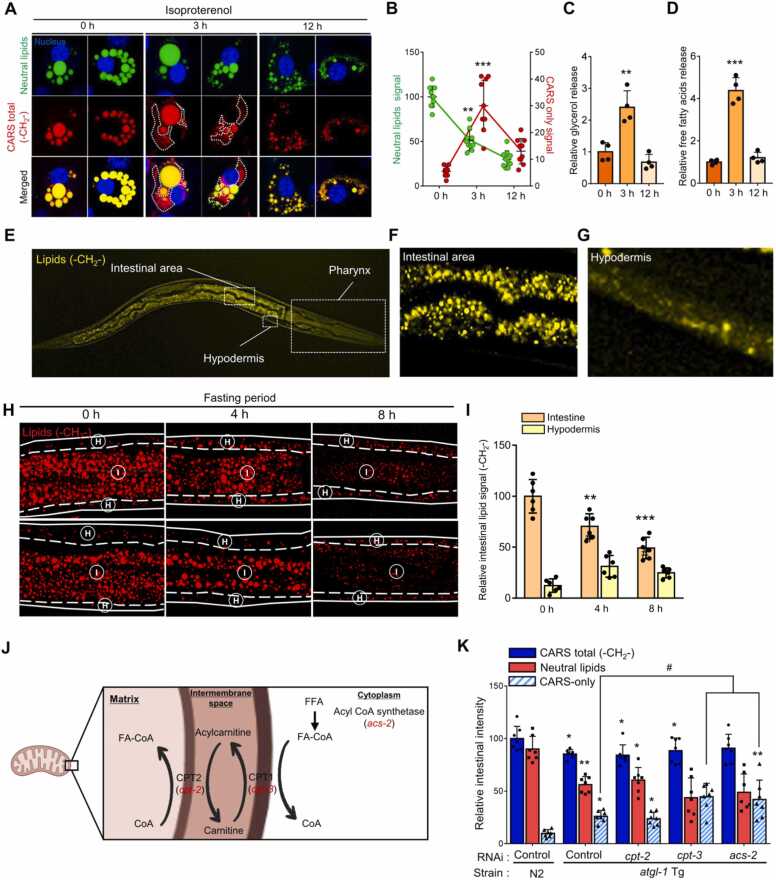
Coherent anti-Stokes Raman spectroscopy (CARS)-only signals detect FFAs in *Caenorhabditis elegans.* (A and B) CARS signal for lipids (-CH_2_-) and neutral lipids dye (BODIPY) signals were imaged together in 3T3-L1 adipocytes treated with isoproterenol (1 μM). White dashed lines indicate CARS-only signals (A). CARS-only signals increased after 3 hours of isoproterenol treatment (B). (C and D) Biochemically measured glycerol and free fatty acid (FFA) levels in isoproterenol-treated 3T3-L1 adipocytes. Similar to the CARS-only signals, both glycerol (C) and FFAs (D) increased after 3 hours of isoproterenol treatment. (E) CARS image of lipids in fed worms. (F and G) Inset images of the intestinal area (F) and hypodermis (G). (H) CARS image of lipids (red) in the anterior intestine during fasting. Hypodermal area “H” around the intestinal area between dashed lines, “I” between solid and dashed lines. (I) Unlike the hypodermis, the intestine showed a decrease in stored lipids over time during fasting (*n* = 6). (J) Conserved fatty acid oxidation-related genes (red characters). (K) In *atgl-1* Tg worms, CARS-only signals were increased and further enhanced when fatty acid oxidation genes were suppressed via RNAi. Data represent the mean ± SD; *P < .05, **P < .01, ***P < .001 vs 0 hour isoproterenol treatment (B-D), vs N2 control RNAi (N2 Con RNAi) (K), ^#^*P* < .05.

To further test whether such quantitative changes in FFAs from whole-worm extracts occur in the anterior intestinal area, nonoverlapping signals between CARS (whole aliphatic lipids) and neutral lipid dyes were carefully scrutinized. Upon isoproterenol treatment, which induces beta-adrenergic stimulation, the “CARS-only signals” were increased, accompanied by biochemical measurements of lipolytic products in differentiated 3T3-L1 adipocytes (Fig. 3A-D). These data suggest that the CARS-only signals represent FFAs generated by the breakdown of stored neutral lipids during lipolysis. To validate whether the CARS-only signals represented FFAs in *C. elegans*, we observed the CARS signal throughout *C. elegans* (Fig. 3E). CARS signals were detected mainly in the intestinal area and hypodermis (Fig. 3F and G), and anterior intestinal CARS signals were decreased after fasting (Fig. 3H and I), as observed using neutral lipid staining. When well-conserved fatty acid oxidation-related genes (Fig. 3J) were suppressed by RNAi in N2 wild-type, and *atgl-1* overexpressed transgenic (*atgl-1* Tg) worms, the level of CARS-only signals was elevated in *atgl-1* Tg worms and was further enhanced by *cpt-3* and *acs-2* RNAi (Fig. 3K), indicating that CARS-only signals represent lipolysis-derived FFAs.

**Fig. 4:**
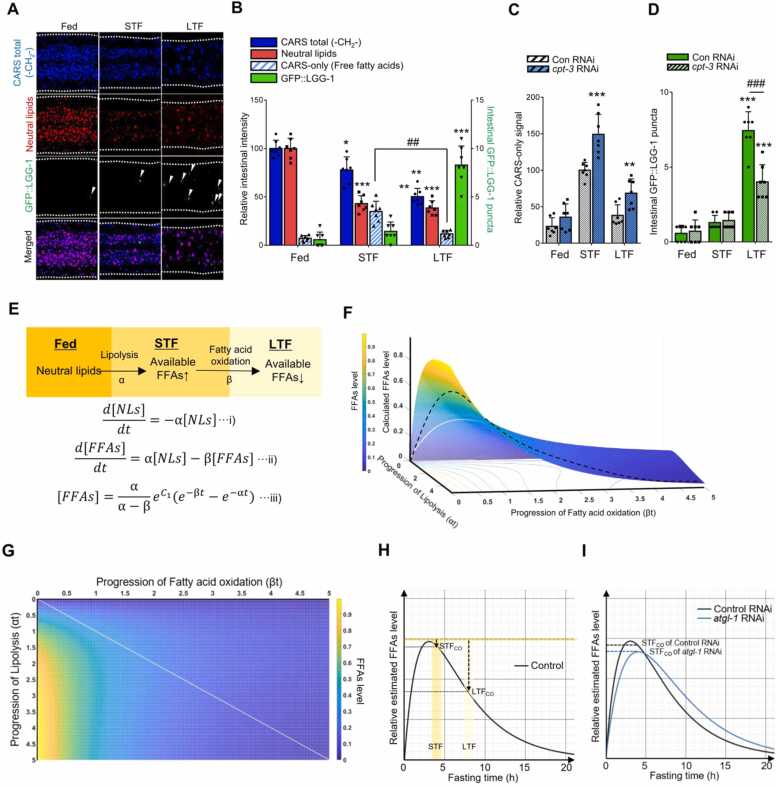
Oxidation of lipolysis-derived free fatty acids (FFAs) is required for autophagy initiation. (A) Coherence anti-Stokes Raman spectroscopy (CARS) signals for lipids (-CH_2_-) and neutral lipid dye (LipidTOX) were monitored together with GFP::LGG-1 puncta (white arrowhead) during fasting (short-term fasting [STF] [4 hours], long-term fasting [LTF] [8 hours]). (B) CARS-only signals increased during STF, followed by a decrease during LTF when GFP::LGG-1 puncta were increased (STF [4 hours], LTF [8 hours]). (C) CARS-only signals of *cpt-3* RNAi worms upon fasting (STF [4 hours], LTF [8 hours]). (D) GFP::LGG-1 puncta of *cpt-3* RNAi worms upon fasting (STF [4 hours], LTF [8 hours]). (E) FFA levels were calculated upon the progression of lipolysis and fatty acid oxidation. Nonlinear equations were obtained (i and ii), where α and β are the elimination rate constant of neutral lipids via lipolysis and FFAs via oxidation, respectively. (iii) was obtained from (i and ii). (F) Progression of lipolysis and fatty acid oxidation are αt and βt, respectively (dashed line represents changes in FFA levels over time that can be determined according to α and β). (G) Top view of (F). (H) Graph for estimated FFA levels when α and β were 0.4909 and 0.3736, respectively, calculated from the mean value of CARS-only signals observed in STF (STF_CO_) and LTF (LTF_CO_). (I) Estimated FFA levels in *atgl-1* RNAi and control group. Data represent the mean ± SD; *P < .05, **P < .01, ***P < .001 vs fed group, and ^##^*P* < .01, ^###^*P* < .001.

In line with the pattern of fatty acids from the metabolomic analysis data, CARS-only signals were increased in STF worms, whereas the extent of neutral lipid dye signals in the anterior intestinal area rapidly decreased in fasted worms, indicating that lipolysis-derived FFAs were potently increased during STF (Fig. 4A). In LTF worms, CARS-only signals decreased in LTF worms, accompanied by considerably enhanced GFP:LGG-1 puncta (Fig. 4A and B). Autophagy initiation decreased when the catabolic breakdown of FFAs was repressed by *cpt-3* knockdown (Fig. 4C and D), suggesting that autophagy is initiated after consuming lipolysis-derived FFAs during LTF.

### Autophagy Is Initiated With a Decrease in Lipolysis-Derived FFA Levels

To determine whether a decrease in lipolysis-derived FFA levels, rather than the amount of remnant FFAs, acts as a prerequisite for autophagy initiation, a time kinetic model to estimate the levels of FFAs during lipid catabolism must be established. Based on the sequential processes of lipolysis and fatty acid oxidation, the rate of FFA change is likely determined by neutral lipid degradation via lipolysis and fatty acid oxidation (Fig. 4E). The graph of the time kinetic model to estimate FFA levels (Fig. 4F) suggested that FFAs levels were largely increased by lipolysis progression and decreased by fatty acid oxidation (Fig. 4G). The estimated FFAs levels on the surface of the graph, designated by the values of the CARS-only signals in STF (STF_CO_) and LTF (LTF_CO_) (Fig. 4F, dashed line, CO corresponds to CARS-only signals), were obtained, as shown in Fig. 4H. In addition, FFA levels decreased from the maximum FFA peak (Fig. 4H, dashed orange line) arising from lipolysis and were greater in LTF than in STF (Fig. 4H, dashed black arrows), indicating that lipolysis-derived FFAs were gradually oxidized after STF. In *atgl-1* RNAi worms, the lipolysis rate was set to “low.” FFA levels in *atgl-1* RNAi worms in the STF were expected to be lower than those in controls (Fig. 4I). Moreover, the estimated FFA level pattern in *atgl-1* RNAi indicated that the amount of FFAs that decreased from the maximum FFA peak was lower than that in control RNAi during fasting. Thus, the delay in autophagy initiation in the *atgl-1* RNAi group (Fig. 2F) was likely due to a slight decrease in lipolysis-derived FFA levels. The estimated levels of FFAs obtained from the nonlinear kinetic model and experimental values suggest that consumption of lipolysis-derived FFAs, rather than remaining FFAs, is crucial for autophagy initiation.

### Activated Protein Kinase A Attenuates AMPK-Dependent Autophagy Initiation During STF

**Fig. 5:**
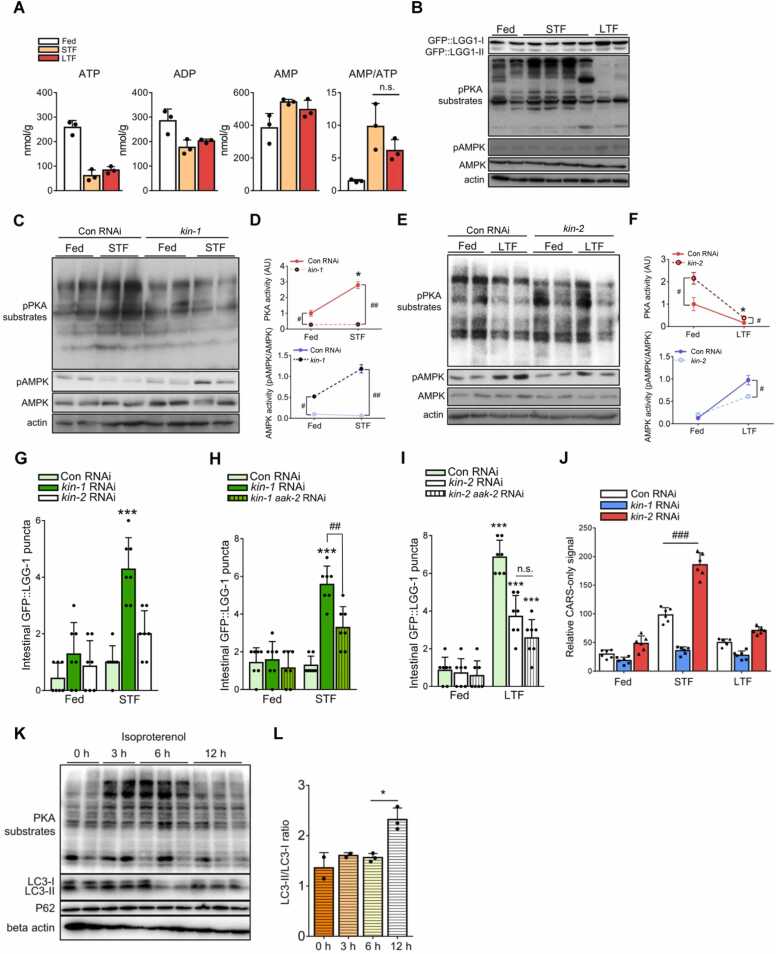
Protein kinase A (PKA) is activated during short-term fasting (STF) and suppresses AMPK-dependent autophagy. (A) Ratio of AMP/ATP levels from metabolomic analysis (STF [4 hours], long-term fasting [LTF] [8 hours]). (B) Western blot of protein extracts from worms during fasting (STF [4 hours], LTF [8 hours]). (C) Western blot of protein extracts from *kin-1* RNAi worms during fasting (STF, 4 hours). (D) Quantification of PKA activity (pPKA substrates normalized to the fed group) and AMPK activity (pAMPK normalized to AMPK in each group). (E) Western blot of protein extracts from *kin-2* RNAi worms during fasting (LTF, 8 hours). (F) Quantification of PKA activity (pPKA substrates normalized to the fed group) and AMPK activity (pAMPK normalized to AMPK in each group). (G) *kin-1* RNAi worms showed increased GFP::LGG-1 puncta during STF (*n* = 7). (H and I) Double RNAi knockdown with *aak-2* decreased GFP::LGG-1 puncta during fasting (STF [4 hours], LTF [8 hours]) (*n* = 7). (J) Upregulation of PKA activity by *kin-2* RNAi resulted in increased FFA levels during STF (STF [4 hours], LTF [8 hours]). (K) Western blots of isoproterenol-treated (1 μM) 3T3-L1 adipocytes, PKA activity decreased after 12 hours of isoproterenol treatment, which is the time point at which CARS-only signals decrease (Fig. 4B). (L) LC3-II/LC3-I ratio increased when PKA activity and CARS-only signals decreased. Data represent the mean ± SD; *P < .05, ***P < .001 vs fed and ^#^*P* < .05, ^##^*P* < .01. ns, not significant.

The decrease in lipolysis-derived FFA levels followed by autophagy initiation prompted us to investigate the key signaling pathway(s) involved in this regulation. Considering that there was no difference in the AMP/ATP ratio between STF and LTF (Fig. 5A) and that autophagy initiation predominantly occurred during LTF, we hypothesized that a suppressive mechanism(s) of autophagy initiation occurs during STF. Given that protein kinase A (PKA) and AMPK are well-known for lipid breakdown and autophagy initiation during fasting (Carmen and Víctor, 2006, Hardie, 2008), respectively, we examined the time course of PKA and AMPK activation during fasting. In worms, the levels of phospho-PKA substrates rapidly increased and decreased during STF and LTF, respectively, whereas those of phospho-AMPK appeared to increase during LTF (Fig. 5B), indicating that the enzymatic activity of AMPK was upregulated later than that of PKA. Based on these results, we hypothesized that PKA inhibits AMPK during STF, thereby suppressing autophagy. To further this result, worms were subjected to RNAi against *kin-1* and *kin-2*, which are conserved genes of the mammalian PKA catalytic and regulatory subunits, respectively (Reimann et al., 1971). Compared with worms in the control group, worms in the *kin-1* RNAi group showed decreased levels of phospho-PKA substrates during STF, whereas those of phospho-AMPK were increased (Fig. 5C and D). In contrast, worms in the *kin-2* RNAi group showed increased levels of phospho-PKA substrates during LTF, whereas those of phospho-AMPK were decreased (Fig. 5E and F). Moreover, *kin-1* RNAi worms showed increased GFP:LGG-1 puncta signals even during STF, suggesting that PKA activity during STF suppresses AMPK activity and autophagy initiation (Fig. 5G). To further examine whether PKA inhibits autophagy initiation via AMPK, the AMPK catalytic subunit gene *aak-2* (Lee et al., 2008) was suppressed. As indicated in Fig. 5H, the increased number of GFP::LGG-1 puncta in the *kin-1* RNAi group was downregulated by *kin-1* and *aak-2* double RNAi. However, there was no significant difference in GFP::LGG-1 puncta between the *kin-2* single RNAi group and *kin-2*, *aak-2* double RNAi group during LTF (Fig. 5I). In addition, elevated CARS-only signals in the *kin-1* RNA group during STF were observed, suggesting that intracellular FFA levels and PKA activity are temporally coupled for autophagy suppression (Fig. 5J and Fig. S2A-C). To investigate the potential relationship among intracellular FFA levels, PKA activity, and autophagy initiation in mammalian cells, 3T3-L1 adipocytes were treated with isoproterenol. Similar to the results observed in worms, isoproterenol-treated 3T3-L1 adipocytes quickly elevated and then decreased the levels of phospho-PKA substrates (Fig. 5K). When the level of phospho-PKA substrates decreased, the autophagy initiation marker LC3-II/LC3-I ratio increased (Fig. 5K and L). Considering these data from worms and mammalian adipocytes, there may be an evolutionarily conserved temporal relationship between intracellular FFA levels, PKA activity, and autophagy initiation. These data suggest that activated PKA during STF treatment represses autophagy initiation via AMPK inhibition for the preferential use of lipolysis-derived FFAs (Fig. S2D).

### Hyperactive Autophagy Reduces Food Foraging Efficiency and Lifespan in Worms

**Fig. 6:**
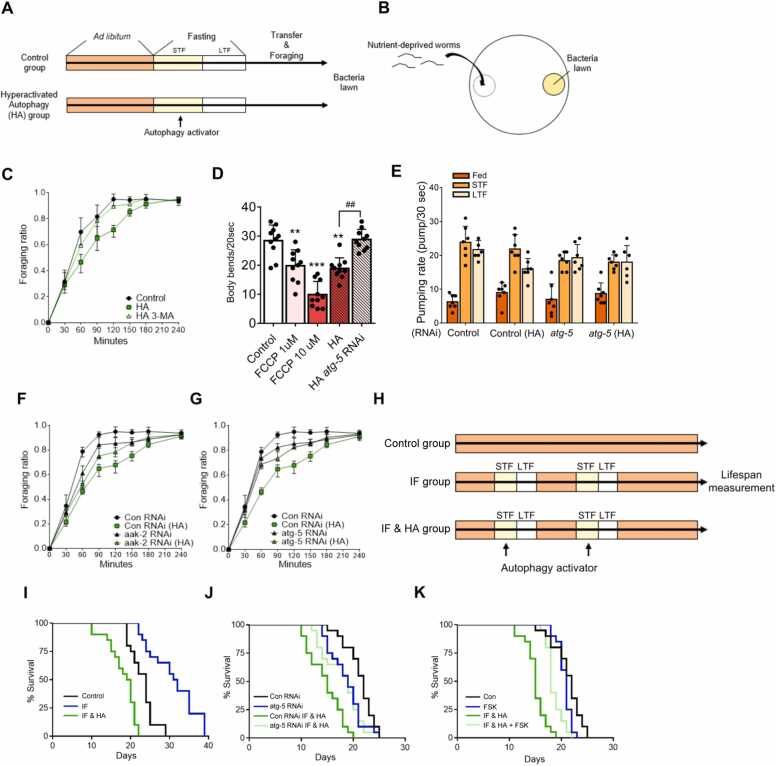
Hyperactive autophagy decreases food foraging behavior and lifespan. (A) Worms were treated with AMPK activator berberine (BBR) to induce hyperactive autophagy (HA) during short-term fasting (STF), followed by food foraging behavior measurement (STF [4 hours], long-term fasting [LTF] [8 hours]). (B) Foraging ratio was measured over time as the number of worms on bacteria lawn among the transferred nutrient-deprived worms. (C, F, and G) Foraging ratio measurement (STF [4 hours], LTF [8 hours]) (*n* = 4 biological replicates of 20 worms per experimental condition). 3-Methyladenine (3-MA) was used as an autophagy inhibitor. (D) Bending rate of worms (*n* = 10). FCCP, trifluoromethoxy carbonylcyanide phenylhydrazone. (E) Pumping rate of worms (*n* = 7). (H) Worms were treated with BBR to promote HA during STF, followed by lifespan measurement. IF, intermittent fasting (STF [4 hours], LTF [8 hours]). (I-K) Survival rate measurement (*n* > 50 for each condition). FSK, forskolin. Worms were treated with BBR (100 μM), 3-MA (10 μM), and FSK (200 μM) for 1 h. Data represent the mean ± SD; **P < .01, **P < .001 vs control and ^##^*P* < 0.01.

To examine the biological importance of PKA-mediated autophagy suppression during STF, worms were treated with the AMPK activator berberine (Kim et al., 2016, Li et al., 2015) to induce enhanced autophagic activity, known as hyperactive autophagy (HA) (Fig. 6A). When the control and HA groups were transferred to agar spotted with bacteria after fasting (Fig. 6B), the HA group exhibited decreased foraging behavior (Fig. 6C). As the bending rate was decreased by treatment with the mitochondrial oxidative phosphorylation uncoupler trifluoromethoxy carbonylcyanide phenylhydrazone, the HA group showed a decreased bending rate (Fig. 6D, Supplemental Videos 1-5). Considering that the HA group showed a similar pattern of an increased pumping rate (Fig. 6E, Supplemental Videos 6 and 7), the decreased foraging behavior may have been due to a decrease in locomotor activity rather than sensing the food source.

Unexpectedly, these decreased foraging behaviors were rescued to a certain extent by *aak-2* or *atg-5* RNAi, indicating that AMPK-dependent autophagy initiation during STF is important for decreased foraging behavior (Fig. 6F and G). To further evaluate whether the deleterious effects of HA influence lifespan, HA was repeatedly induced during intermittent fasting, a well-known condition that prolongs lifespan (Hansen et al., 2008, Lee et al., 2006) (Fig. 6H). Consequently, HA significantly shortened the extended lifespan of the intermittent fasting group (Fig. 6I), which was reversed by *atg-5* RNAi (Fig. 6J). Consistent with the above findings, PKA activator treatment rescued the shortened lifespan of the HA group (Fig. 6K), indicating that PKA activity is pivotal for a normal lifespan by suppressing futile autophagy initiation during STF.

## DISCUSSION

Autophagy is initiated by sensing a low-energy state due to nutrient deprivation (Brooks et al., 2009, Russell et al., 2014, Sakata et al., 2018). Although nutrient availability is a key determinant of autophagy initiation, the mechanism(s) underlying the perception of nutritional scarcity by which cells timely turn on autophagy as the last self-destructive process for energy supply remains unclear. Our results suggest that PKA is crucial for suppressing autophagy initiation during STF when available FFAs are present as energy sources. Several lines of evidence support this hypothesis. First, the degree of PKA activity and intracellular FFAs from lipolysis were elevated during STF when autophagy had not yet occurred. Second, the blockade of fasting-induced lipolysis or fatty acid oxidation resulted in delayed autophagy initiation. Third, activated PKA potently inhibited AMPK-dependent autophagy in STF. In addition, PKA-mediated autophagy suppression during STF prevented deleterious effects such as decreased food foraging and shortened lifespan at the organismal level. Our findings suggest that PKA-mediated lipid breakdown is a crucial safeguarding mechanism against detrimental autophagy initiation in STF.

Although many studies showed that deprivation of amino acids, glucose, and other nutrients activates autophagy (Efeyan et al., 2013, Galluzzi et al., 2014, Kim et al., 2013, Ye et al., 2010), the suppression of autophagy when lipids become available over time under fasting conditions has not been widely examined. Although the lipid content and autophagy signaling in worms have been reported (Kaššák et al., 2020), the previous study did not thoroughly explore the physiological conditions related to lipid availability. Our metabolomic and CARS imaging analyses revealed that alterations in lipolysis-derived FFA levels negatively correlated with autophagy initiation during nutrient deprivation in worms and mammalian adipocytes. Similar to our findings, a previous study showed that autophagy is induced in the liver several hours after serum insulin is decreased, which inhibits lipolysis (Ezaki et al., 2011). Furthermore, at least in worms, the levels of certain glycolysis intermediates and amino acids were not significantly different between STF and LTF or were even higher in LTF (Fig. S1A). These data imply that a reduction in the levels of available FFAs is important for autophagy under physiological fasting conditions. Considering that FFAs have been implicated in several autophagic pathways (Piccolis et al., 2019), future studies of whether increased FFAs during STF can be utilized as energy-producing nutrient sources are required to clarify the role of lipolytic products in autophagy suppression.

PKA and AMPK have been extensively investigated as key energy sensors in response to nutrient deprivation (Carmen and Víctor, 2006, Hardie et al., 2012). PKA and AMPK play different regulatory roles in lipid metabolism. For example, PKA actively catalyzes fasting-induced lipolysis and upregulates intracellular FFA levels, whereas AMPK attenuates these processes (Daval et al., 2005). Moreover, AMPK is activated due to lipolysis and inhibits further lipolysis (Gauthier et al., 2008), supporting the idea that PKA and AMPK crosstalk for balanced energy homeostasis. In the current study, we observed that PKA and AMPK activities were negatively correlated during fasting. Given that PKA activity precedes AMPK during periods of nutrient deprivation, our worm studies revealed that inhibition of PKA activity during STF suppressed lipolysis, leading to increased levels of phospho-AMPK and increased LC3 puncta formation, indicating that PKA influences autophagy initiation during STF by inhibiting AMPK. Altogether, our findings suggest that PKA efficiently orchestrates catabolic pathways for the appropriate transition from lipid usage to autophagy initiation in response to changes in cellular energy levels. However, autophagy can be regulated through intertissue interactions (Bujak et al., 2015, Chen et al., 2023, Wang et al., 2022, Wei Wu et al., 2012). Thus, future studies using more tissue-specific approaches would help further elaborate on these mechanisms.

Given that chronic or HA is closely linked to detrimental outcomes (Bonaldo and Sandri, 2013, Kang et al., 2007, Liu and Levine, 2015, Malicdan et al., 2008, Yamamoto et al., 2018), suppression of improper autophagy initiation during STF may be advantageous for maintaining physiological conditions. Our data showed that autophagy initiation during STF resulted in a decreased bending rate, foraging behavior, and lifespan extension, suggesting that PKA is protective against futile autophagy initiation during STF.

In conclusion, we identified the mechanisms and physiological significance of autophagy suppression during the early phase of fasting. Comprehensive analyses of metabolomics, genetically modified animals, and lipids imaging would provide insights into the preferential utilization of lipolysis-derived FFAs as energy sources before autophagy initiation. Collectively, our data suggest that the sophisticated regulation of lipid catabolism and autophagy through the cooperation between PKA and AMPK is a key process in regulating intracellular catabolism under fasting conditions.

## Author Contributions

Y.J. conceptualized the study, performed the experiments, analyzed the data, and wrote the manuscript. Y.G.J. performed the metabolic analyses. W.T.L. performed cell culture experiments. Y.G.J., W.T.L., J.S.H., K.C.S., and J.Y.H. discussed the study and wrote the manuscript. J.B.K. supervised the study and drafted the manuscript.

## Declaration of Competing Interests

The authors declare that they have no known competing financial interests or personal relationships that could have appeared to influence the work reported in this paper.

## Appendix A Supplemental material

**Supplementary Fig. 1.** Changes in metabolites and lipid metabolism-related gene expression in *Caenorhabditis elegans* during fasting. (A) Levels of carbohydrates related to glycolysis and amino acids from metabolomic analysis (short-term fasting [STF] [4 hours], long-term fasting [LTF] [8 hours]). (B) Reverse transcription-quantitative polymerase chain reaction analysis of lipolysis, lipogenesis, and fatty acid oxidation-related genes after fasting (STF [4 hours], LTF [8 hours]). Data represent the mean ± SD; **P* < .05, ***P* < .01, ****P* < .001 vs fed group, and ^#^*P* < .05, ^##^*P* < .01, ^###^*P* < .001.

.

**Supplementary Fig. 2.** Protein kinase A (PKA) activity and free fatty acids (FFAs) are temporally coupled during fasting. (A-C) Coherent anti-Stokes Raman spectroscopy (CARS) signals for lipids (-CH_2_-) and neutral lipid dye signals (BODIPY) were imaged during fasting (short-term fasting [STF] [4 hours], long-term fasting [LTF] [8 hours]). (D) Increased PKA during STF can break down lipids via lipolysis, allowing the preferential utilization of elevated free fatty acids (FFAs) as energy sources during STF. Data represent the mean ± SD; **P* < 0.05, vs 0 hour isoproterenol treatment and ^###^*P* < .001.

.

**Table S1.** Metabolomic data and comparative analysis of *Caenorhabditis elegans* under different nutritional states. This supplementary table provides a comprehensive overview of the metabolomic profiles and comparative analyses of *C. elegans* under different nutritional conditions: Fed, Fasted 1, and fasted 2. Data were collected from 9 samples using capillary electrophoresis time-of-flight mass spectroscopy (CE-TOF-MS) and liquid chromatography (LC)-TOF-MS in cationic, anionic, positive, and negative modes. The table includes sample details, such as the amounts used, group classifications, and dilution factors for each analytical mode. Relative peak areas were calculated and statistically compared between the different nutritional states, showing mean values, standard deviations, ratios, and *P*-values. Quantitative estimation of specific metabolites is provided, listing the concentrations in nmol/g, along with PubChem CID and HMDB IDs for reference. The table includes data for principal component analysis scores, factor loadings, heat maps, and information on pathway clustering and HMDB categorization, offering insights into the metabolic changes and pathway interactions influenced by the nutritional status of the worms.
