# Supplementary material for: PKA regulates autophagy through lipolysis during fasting
Source: Mol Cells. 2024 Nov 13;47(12):100149. doi: 10.1016/j.mocell.2024.100149 (PMC11697058; doi:10.1016/j.mocell.2024.100149)
Supplement: Supplementary file 2 — Supplementary Fig. 2. Protein kinase A (PKA) activity and free fatty acids (FFAs) are temporally coupled during fasting. (A-C) Coherent anti-Stokes Raman spectroscopy (CARS) signals for lipids (-CH2-) and neutral lipid dye signals (BODIPY) were imaged during fasting (short-term fasting [STF] [4 hours], long-term fasting [LTF] [8 hours]). (D) Increased PKA during STF can break down lipids via lipolysis, allowing the preferential utilization of elevated free fatty acids (FFAs) as energy sources during STF. Data represent the mean ± SD; *P < 0.05, vs 0 hour isoproterenol treatment and ###P < .001. [file mmc2.pdf]

editage

Editing Certificate

This document certifies that the manuscript listed below has been edited to ensure language and grammar accuracy and is error free in these aspects. The edit was performed by professional editors at Editage, a brand of Cactus Communications. The author's core research ideas were not altered in any way during the editing process. The quality of the edit has been guaranteed, with the assumption that our suggested changes have been accepted and the text has not been further altered without the knowledge of our editors.

MANUSCRIPT TITLE

Balancing Lipolysis and Autophagy: PKA as a Key Regulator During Nutrient Deprivation

AUTHORS

Yul Ji, Yong Geun Jeon, Won Taek Lee, Ji Seul Han, Kyung Cheul Shin, Jin Young Huh, and Jae Bum Kim

ISSUED ON

November 05, 2024

JOB CODE

XVEBU_32

THE EDITAGE Promise QUALITY GUARANTEE

Prabh Grewal
Senior Vice President - Editage

editage | helping you get published

Since 2002, Editage has helped over 430,000 authors publish around 1.2 million research papers in scholarly journals across over 1000 disciplines through editorial, translation, transcription, and publication support services. Editage is a brand of Cactus Communications (cactusglobal.com), a science communication and technology company.

CACTUS.

GLOBAL :
+1(833) 979-0061 | request@editage.com

KOREA :
1533-6413 | submit-korea@editage.com
