# Supplementary material for: PKA regulates autophagy through lipolysis during fasting
Source: Mol Cells. 2024 Nov 13;47(12):100149. doi: 10.1016/j.mocell.2024.100149 (PMC11697058; doi:10.1016/j.mocell.2024.100149)

Figure 6. Hyperactive autophagy decreases food foraging behavior and lifespan

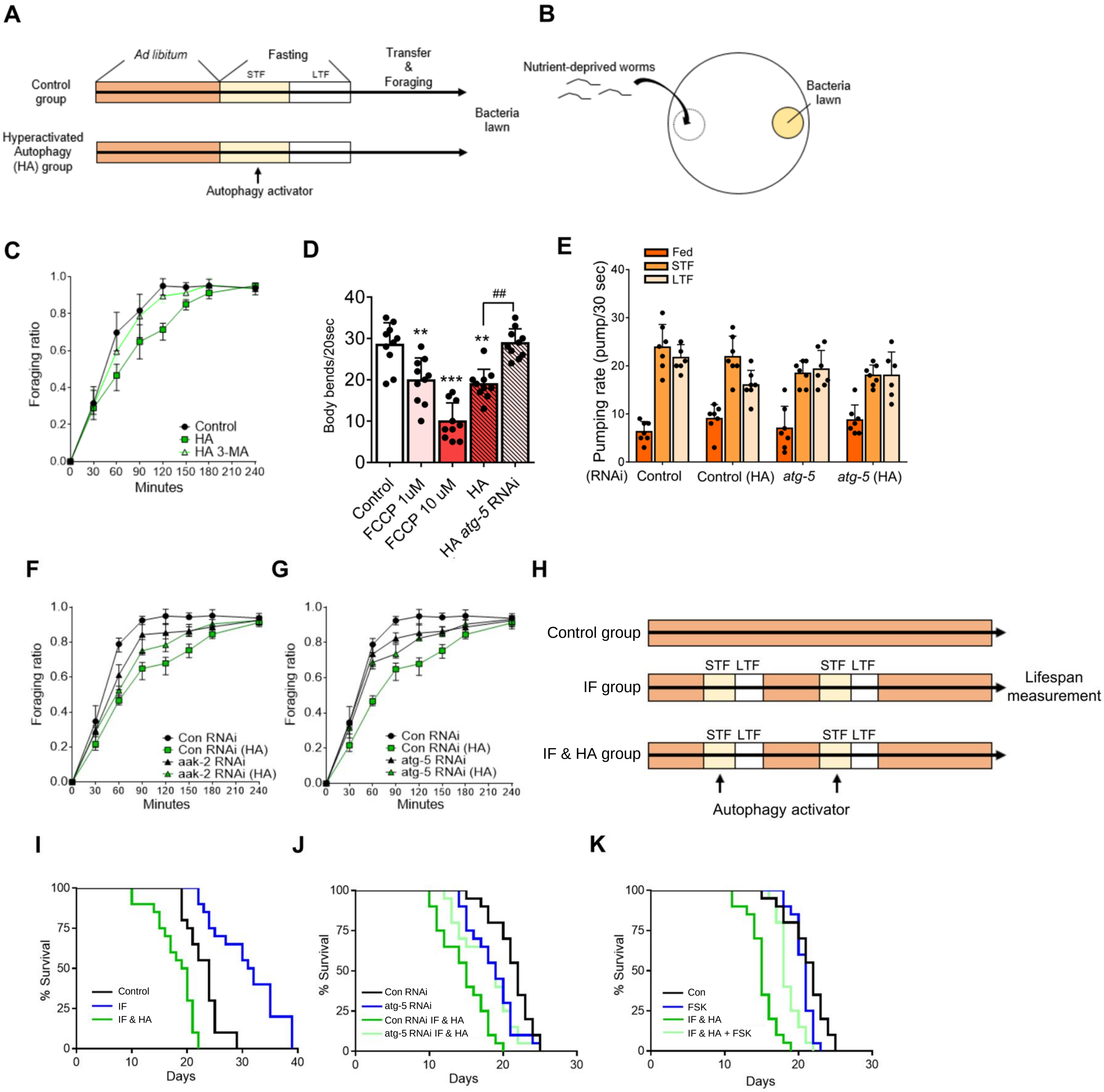

Supplementary Figure 1. Changes in metabolites and lipid metabolism-related gene expression in *C. elegans* during fasting

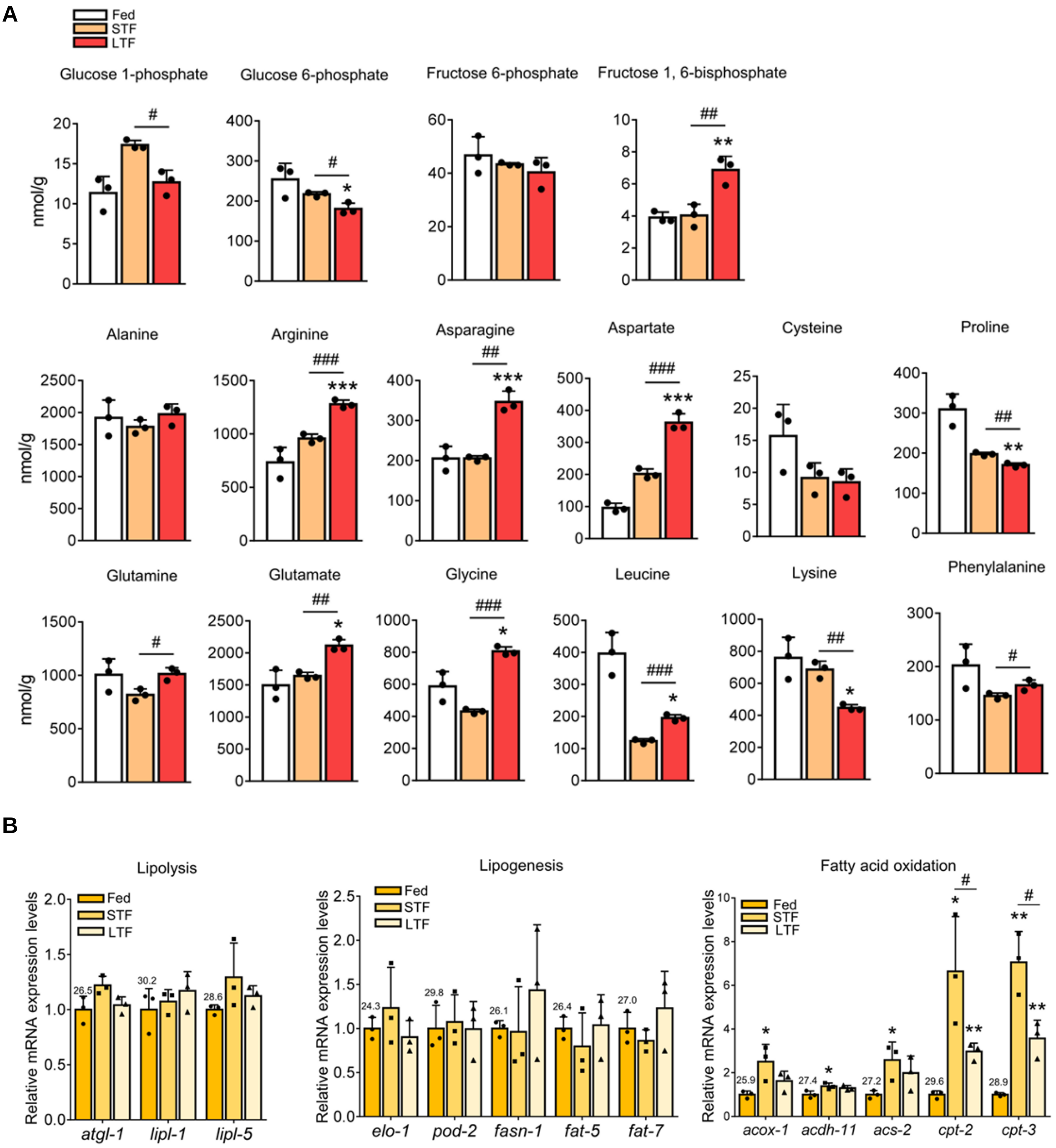

Supplementary Figure 2. PKA activity and FFAs are temporally coupled during fasting

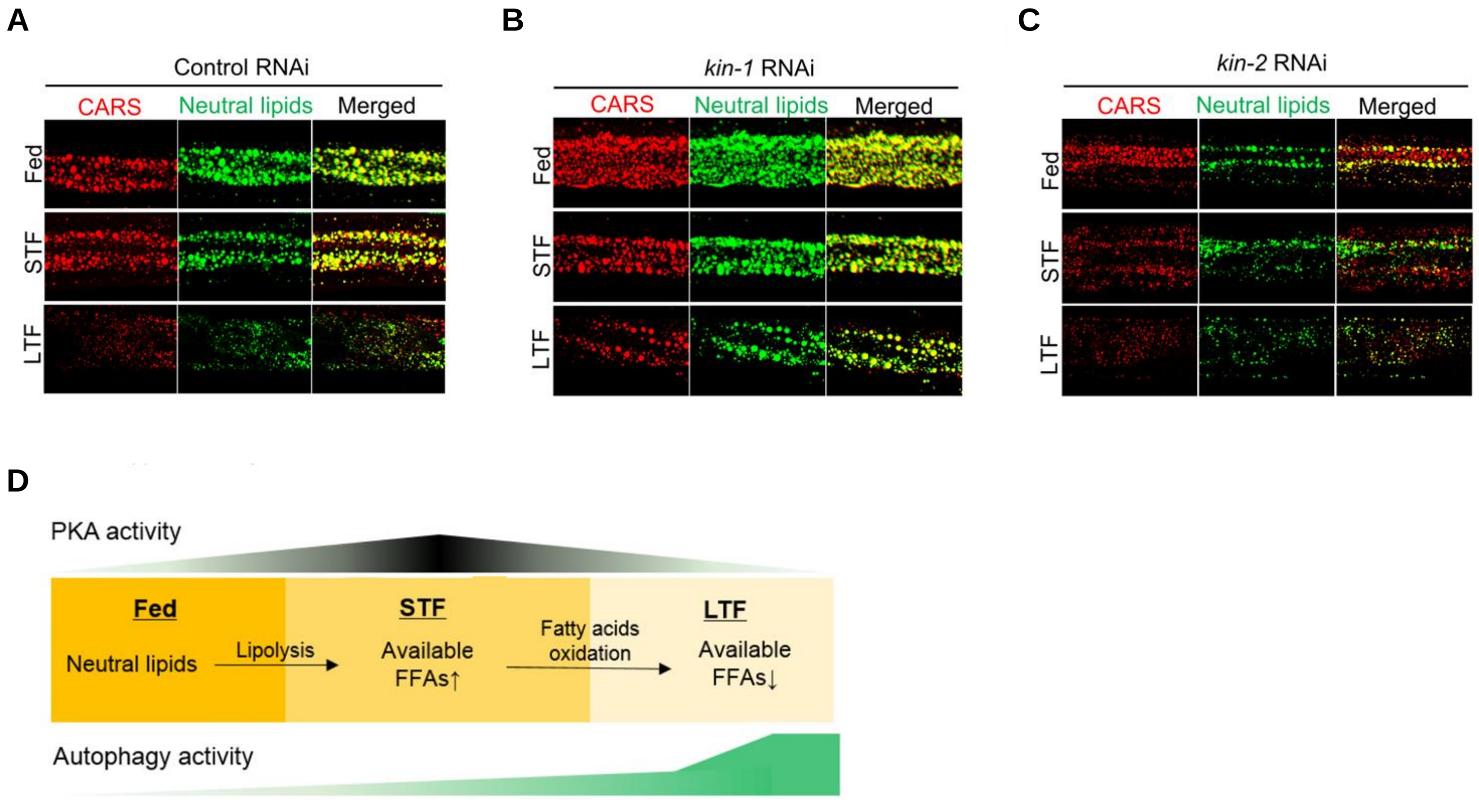

Supplement: Supplementary file 3 — Table S1. Metabolomic data and comparative analysis of Caenorhabditis elegans under different nutritional states. This supplementary table provides a comprehensive overview of the metabolomic profiles and comparative analyses of C. elegans under different nutritional conditions: Fed, Fasted 1, and fasted 2. Data were collected from 9 samples using capillary electrophoresis time-of-flight mass spectroscopy (CE-TOF-MS) and liquid chromatography (LC)-TOF-MS in cationic, anionic, positive, and negative modes. The table includes sample details, such as the amounts used, group classifications, and dilution factors for each analytical mode. Relative peak areas were calculated and statistically compared between the different nutritional states, showing mean values, standard deviations, ratios, and P-values. Quantitative estimation of specific metabolites is provided, listing the concentrations in nmol/g, along with PubChem CID and HMDB IDs for reference. The table includes data for principal component analysis scores, factor loadings, heat maps, and information on pathway clustering and HMDB categorization, offering insights into the metabolic changes and pathway interactions influenced by the nutritional status of the worms. [file mmc3.pdf]
